# The Functional Self: The Minimal Self-Concept Is Protected Against Negative Content

**DOI:** 10.1027/1618-3169/a000582

**Published:** 2023-06-13

**Authors:** Sarah Schäfer, Dirk Wentura, Tarini Singh, Christian Frings

**Affiliations:** ^1^ Cognitive Psychology Unit, University of Trier, Germany; ^2^ Department of Psychology, Saarland University, Saarbrücken, Germany

**Keywords:** self-concept, minimal self, functionality, cognitive processes, self-protection

## Abstract

**Abstract.** Current research describes a particular component of the self-concept that influences a wide variety of cognitive processes while it depicts a rather basic component of the self-concept. However, this *minimal* self seems to be anything but *simple*; in fact, it seems to be highly functional. Based on previous findings on newly formed self-associations, we put the postulated functionality of this minimal self to another test by retesting its protection mechanisms against negative content. In a pilot experiment, we did not find an overall reduction of negative self-assignments against neutral self-assignments. However, the results indicated an initial difference (as hypothesized) between negative and neutral self-assignments, which decreases over the course of the experiment. We put this interactive effect of valence and block to test in our main experiment, which replicated the data pattern of the pilot experiment. In sum, the results indicate a mandatory integration of stimuli into the self-concept and also a reduction of the integration due to negative valence, thereby supporting a robust protection mechanism.


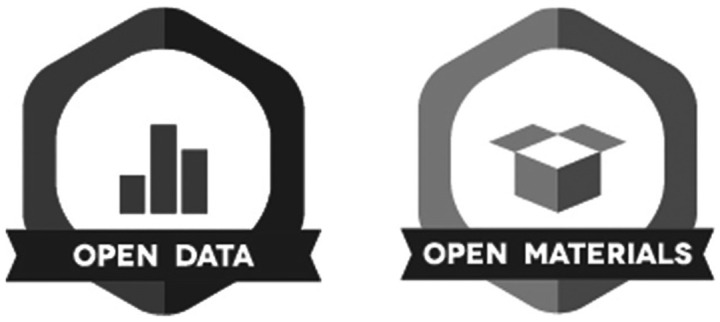


Our self-concept has far-reaching impact as it influences the way we perceive and process our surroundings, plays an important role in our psychological development throughout the whole life span, and determines our social interactions as well as our mental health. Consequently, the self-concept constitutes a basic issue in psychological research. The famous cocktail party effect (Conway et al., 2001; Moray, 1959; Shapiro et al., 1997) and similar results in different other paradigms (Alexopoulos et al., 2012) as well as various neurophysiological studies (e.g., Gray et al., 2004; Hu et al., 2016) suggest that stimulus processing is different when the stimulus is self-relevant than when it is non-self-relevant. However, a concrete definition and a lot of research are necessary to understand the way in which we are influenced by self-relevant stimuli in our surroundings.

A cognitive-psychological approach differentiates between various components of the self-concept. One component of the self is defined as an immediate, rather unconscious, simple differentiation between self and nonself, which is based on fundamental brain processes and on body perception (Ehrsson et al., 2004; Gallagher, 2000; Northoff, 2016). This basic self-concept is typically called the *minimal* self (Gallagher, 2000; Hommel, 2019) and can be distinguished from a more elaborate self-image, the *narrative* self, which is influenced by what we and others think and tell us about ourselves (Gallagher, 2000; Schäfer & Frings, 2019b). To investigate the basic (rather intern) component of the self, a focus on simple behavioral action effects rather than elaborated decisional biases has been established and a useful tool to test for basic self-effects is the so-called self-prioritization effect (SPE) in the matching paradigm introduced by Sui et al. (2012). In this paradigm, simple shape stimuli are associated with the self and non-self-relevant others by instruction (e.g., “You are the triangle. Your mother is the rectangle. A stranger is the circle”). Subsequently, in a series of trials, pairs of labels and shapes are presented, and participants must quickly affirm (e.g., in case of “I – triangle”) or negate (e.g., in case of “mother – triangle”) the combination. Typically and reliably, participants respond faster and more accurately on the self-associated combinations in comparison to the other-associated combinations – this pattern constitutes the SPE (e.g., Schäfer, Wesslein, et al., 2016; Sui et al., 2012; Woźniak et al., 2018). The underlying processes of this effect are still topic of an ongoing debate (Falbén et al., 2019, 2020; Janczyk et al., 2019; Reuther & Chakravarthi, 2017; Schäfer et al., 2020; Stein et al., 2016). Taken together, the effect is interpreted in terms of an integration of new stimuli into the self-concept (Humphreys & Sui, 2016; Schäfer, Frings, & Wentura, 2016), but little is known about potential moderators of these self-integration mechanisms.

Besides methodological factors such as stimulus concreteness (Wade & Vickery, 2017), grammatical distinctiveness (Schäfer et al., 2017), or the amount of self-relevance of the used stimuli (Golubickis et al., 2020), which seem to influence the results in the matching paradigm, little is known about what influences self-integration. Yet, one factor has been identified in previous studies to determine the amount of self-integration: the particular valence of the to-be-integrated stimuli.

Specifically, two studies revealed that self-integrations as measured by the SPE are protected against negative content. In one study, in a slightly different paradigm, the prioritization of self-assigned positive images was compared to the prioritization of negative poster images (Golubickis et al., 2019). Thus, while neutral stimuli are usually prioritized after they have been presented as objects owned by the self in an object-ownership task (Golubickis et al., 2018), this prioritization was shown to be reduced for negative stimuli (i.e., poster of images which have been rated as undesirable by the participants; Golubickis et al., 2019). In a second study, in which morphed faces with happy or sad expressions were associated with the self (or with non-self-relevant others), self-prioritization was significantly larger when a happy face was to be integrated than when a sad face was to be integrated (Constable et al., 2020). Interestingly, this reduction of the SPE with negative stimuli only occurred when the used face stimuli were strong representations of the emotion; thus, with only slightly sad- or happy-looking faces, self-prioritization was not influenced. Furthermore, the reduction of self-prioritization was also shown for dark versus light stimuli, in line with metaphoric language such as “The bright side of life” and “In a dark place” (Constable et al., 2020). Hence, these studies provide evidence for the assumption that self-integration is significantly influenced by the emotional valence of the content that is to be integrated and that the integration of negative content is prevented.

Considering the influence of the minimal self on the way we perceive and process our surroundings (as mentioned at the beginning), it seems to be essential that this part of the self follows suitable and functional mechanisms. Further research already revealed other aspects of such a *functionality* of the minimal self. For example, it has already been shown that self-associations are formed in a *specific* manner. Thus, if the participant’s self was associated with a feature conjunction (e.g., a red triangle), in a subsequent matching task, the SPE was limited to the entire conjunction (i.e., the red triangle); no prioritization was observed for either feature alone (i.e., red or triangular stimuli, Schäfer, Frings, & Wentura, 2016). That is, the prioritization resulting from the association of a formerly neutral stimulus with the self focuses on that specific stimulus and is not broadened to any similar stimulus. Such a focused prioritization of only the exact stimulus avoids a spreading of cognitive resources to stimuli which are not actually associated with the self (but just share features with the self-associated content). Furthermore, newly formed self-associations were shown to be rather *stable*. For example, in one study, initially created self-associations (and other associations) were later rearranged (e.g., “You have been the triangle, but now you are associated with the square and a friend is associated with the triangle”; Wang et al., 2015). While the first association resulted in a typical SPE, the reversal of the associations resulted in slower response times and lower accuracy in those nonmatching trials involving the previously self-associated stimulus (e.g., “I – triangle”). These findings indicate aftereffects of the previous association with the self, which suggest a stability of self-associations. The opposite, that is, a fast decay of self-associations, would force the system to constantly regenerate associations of relevant content with the self (i.e., a version of the well-known stability–plasticity dilemma; Grossberg, 1980, 1987). Thus, the reported specificity and stability of self-associations support the assumption that self-associations are formed (and maintained) in a functional manner.

Furthermore, the prioritization of a stimulus after it has been assigned with the self was demonstrated to be stronger when the stimulus was assigned with a *good self* (i.e., morally good aspects of the self) than when it was assigned with a *bad self* (Hu et al., 2019), indicating an influence of emotional aspects on self-integration. Comparably, self-prioritization was reduced under negative mood. Specifically, the SPE was reduced (compared to a neutral control condition) after participants read a list of self-related statements that induce negative mood and listened to negative music (Sui et al., 2016). An influence of emotional aspects on self-integration would further support the assumption that integration of stimuli into the minimal self follows functional mechanisms. In that regard, the maintenance of a positive self-concept by virtue of several biases and mechanisms (e.g., Greenwald, 1980; Taylor & Brown, 1988) is one of the main self-protection functions discussed in the broader self-concept literature. For example, the well-documented self-serving attributional bias – that is, people tend to attribute negative events less frequently to themselves (i.e., internal) compared to positive events (e.g., Mezulis et al., 2004) – suggests a protection of the self against negative characteristics. Although such a focus on complex explicit event explanations pertains to aspects of the *narrative self* (see above), this bias might still have its roots at a more basic level. Most relevant in the present context, Kuiper and Derry (1982; Kuiper et al., 1985) found better incidental recall of positive compared to negative words if these words were rated with regard to self in the encoding phase. Specifically, the authors instructed their participants to categorize negative (more specifically, depression-related) and non-negative (i.e., non-depression-related) adjectives either with regard to a semantic feature or with regard to the self (i.e., whether they describe themselves or not). A subsequent incidental recall test showed (for nondepressive participants) a marked lower recall rate for negative compared to non-negative adjectives, but only if they were rated with regard to the self.

We set out to test the strength of the protection mechanism. In two experiments, the prioritization of negatively connoted self-associated stimuli was compared to the prioritization of neutral self-associated stimuli. By avoiding a positive condition, the pure effect of negative valence can be seen. Additionally, the simple design of the experiments allowed to depict the possible effect independent of some previous confounds, which might be relevant. For example, in the study with morphed faces (Constable et al., 2020), emotional valence of the self-associated face was confounded with the emotional valence of the non-self-associated face. Thus, the reduced integration in one condition can be explained by the negative valence of the self-associated stimulus or, in the same manner, by the positive valence in the non-self-associated condition. Furthermore, in the study using the object-ownership task (Golubickis et al., 2019), valence (positive vs. negative) was confounded with arousal (lower vs. higher; while arousal was explicitly tested and reported to be lower for positive than for negative posters). Moreover, whereas our pilot experiment – such as also the studies cited above – used given negative and neutral stimuli to establish the decisive independent variation (i.e., was in fact quasi-experimental), we manipulated valence experimentally by means of evaluative conditioning in our main experiment.

Taken together, the results in previous studies speak in favor of a protection of the minimal self against negative content. Given these first indications, we put the question of a functional minimal self to another test and circumvent the caveats of the previous attempts by using neutral to-be-integrated stimuli and evaluative conditioning.^1^

## The Current Study

In detail, we tested the self-integration of negatively connoted stimuli by associating different stimuli with the self and with others in the matching paradigm (Sui et al., 2012). In a pilot experiment, participants were instructed to learn associations of three shapes of weapon symbols (e.g., a grenade) with one of three labels (one self-relevant and two non-self-relevant). Thus, the to-be-associated shapes in the experimental condition were negatively connoted by their inherent meaning. In a control condition, participants underwent the same procedure with the only exception that the to-be-associated stimuli were neutral geometric shapes. The resulting SPEs in both conditions were compared to each other.

A priori, we postulated a significant reduction of the SPE in the condition with negative stimuli compared to the condition with neutral stimuli. To anticipate, the data did not reveal this effect in the a priori planned analysis. However, in a post hoc analysis, the pilot data made us aware of a meaningful sequence effect: The postulated difference between the SPE for neutral and negative stimuli decreased over the course of the experiment. As the pure effect of negative valence against neutral assignments might be rather weak and as the negative valence of the used stimuli might vanish over the course of the experiment (as explained below), such an interactive effect of valence and experimental blocks did not appear implausible. Hence, we conducted our main experiment to test whether the post hoc revealed data pattern of the pilot experiment could be replicated. In addition to that, in the main experiment, valence was manipulated experimentally. Consequently, this experiment tested the interaction effect with sufficient power and with non-quasi-experimental material. The data pattern was supposed to indicate a reduced SPE with negatively connoted stimuli in the first block, but no such difference in the second block (i.e., an interaction of valence and experimental block).

## Pilot Experiment

The pilot experiment tested for the first time the SPE in the matching paradigm if all to-be-associated stimuli (i.e., the self-associated and the non-self-associated stimuli) were either neutral or negative in a particular condition. Based on the assumption of a functionality of self-associations, we expected the SPE with negative stimuli to be smaller than a typical SPE with neutral stimuli.

### Method

#### Participants

Forty students from the University of Trier (32 female) took part in the experiment receiving course credit. The data of one participant (female) were discarded prior to analysis because he committed far too many errors and responded far too slowly (i.e., far-out value according to Tukey, 1977), indicating that this participant did not follow the instructions. Thus, the total sample size was *N* = 39 (19 in the negative-valence condition). The median age was 21 years (ranging from 18 to 31), and participants had normal or corrected-to-normal vision.

The SPE was rather large in previous studies (*d*_*z*_ > 0.80; Schäfer, Wesslein, et al., 2016; Sui et al., 2012) so that the power to detect the standard SPE (i.e., with neutral materials) was 1 − β > 0.95 (α = 0.05, one-tailed) with *N* = 20 participants (calculations were done with G*Power 3.1.9.4; Faul et al., 2007). The same sample size was used for the SPE with negatively connoted stimuli to obtain a first estimate of this effect. Thus, we consider the pilot experiment as a first exploration of whether there is an indication of a reduced SPE with negatively connoted stimuli (and with the main experiment to test also for smaller effects). Note that we did not plan for a significant interaction test with appropriate power in this pilot study since even a notable difference in effects (e.g., *d* = .40; i.e., halving the SPE) already results in a sample size estimate of *N* = 156 (for power 1 − β = .8, α = 0.05, one-tailed) for this between-participants comparison.

#### Design

The experiment comprised a 2 (stimulus valence: *negative* vs. *neutral*) × 2 (matching condition: *matching* vs. *nonmatching*) × 3 (association: *self* vs. *mother* vs. *acquaintance*) mixed-measures design with valence as a between-subject factor.

#### Material and Apparatus

The experiment was conducted using standard PCs with standard TFT monitors, German QWERTZ keyboards, and the E-Prime 2.0 software. The words were written in Courier New font, and all words and shapes were presented in white on black background. The combinations were presented with the shape above a central fixation cross and a label below the fixation cross. With a viewing distance of about 60 cm throughout the experiment, the presented stimuli subtended 3.3° × 3.3° visual angle for the shapes and 0.6° font height for the labels. We used the German words *Ich* [I] as the self-relevant label and *Mutter* [mother] and *Bekannter* [acquaintance] as the two non-self-relevant labels. A total of six different negative symbols were used as shapes in the negative-valence condition. For each participant, one of six predefined assignments of three symbols, each paired with a label, was randomly chosen; participants in the neutral-valence condition were randomly assigned to one of three shape–label assignments (see Appendix A).

At the beginning of the experiment (i.e., in the association phase; further information in the Procedure section), each symbol was followed by a written label: the German words *Dolch* [dagger], *Morgenstern* [morning star], *Revolver* [revolver], *Granate* [grenade], *Bombe* [bomb], and *Keule* [mace] in the negative-valence condition and *Dreieck* [triangle], *Kreis* [circle], and *Quadrat* [square] in the control condition.

#### Procedure

Participants were seated at individual computers at separate tables (participants were tested parallel in pairs). Instructions were presented on the screen and summarized by the experimenter in the beginning of the experiment. The experiment started with a learning phase, in which the to-be-learned associations were displayed for 60 s. The associations were displayed as follows: “I am the symbol… My mother is the symbol… An acquaintance is the symbol…” (row by row), and after each sentence, a picture of the symbol as well as the associated label was presented. After the learning phase, the matching task began. Participants were instructed to place the index finger of the left hand on the S-key (nonmatching response) and the index finger of the right hand on the L-key (matching response). Each trial started with a 500-ms presentation of a black screen, followed by a fixation cross for 500 ms. Then, a label–shape combination was presented for 100 ms, followed by a black screen until the participant responded or 1,500 ms had elapsed. Participants’ task was to judge whether the displayed combination corresponded to one of the initially learned assignments. One experimental session consisted of a short practice block with 24 trials (in which feedback was presented on the screen) and an experimental block with 300 trials (without feedback). In the experimental phase, each shape was presented in 100 trials and half of the trials depicted matching, half nonmatching combinations. The same proportions were realized in the practice phase. Trials were presented in a random order. Note that nonmatching trials serve as filler trials to make the matching task a reasonable task. Given the intermixed structure of self- and other-associated labels and symbols in each nonmatching condition, no hypotheses are formulated for nonmatching trials.

### Results

Only correct responses with RTs above 200 ms and below 1.5 interquartile ranges above the third quartile of the overall RT distribution (Tukey, 1977) were used for the RT analysis. Averaged across participants, 86% of the trials were selected for RT analysis; 12% of the trials were excluded because of erroneous responses and 4% due to the RT-outlier criteria. Mean RTs and sensitivity measures are shown in Table 1, separately for the first and second blocks of trials in the experiment (for a detailed description of the design, see the following paragraph).

**Table 1:** Mean RTs in ms in matching trials as well as sensitivity in *d*′ in the pilot experiment as a function of stimulus valence, association, and block (standard deviations in parentheses)

Stimulus valence	Stimulus association	RTs		*d*′	
		Block		Block	
		First	Second	First	Second
Negative	Self	717 (86)	669 (58)	3.6 (0.6)	3.5 (0.6)
	Mother	740 (112)	715 (87)	3.6 (0.8)	3.6 (0.8)
	Acquaintance	822 (124)	790 (88)	3.0 (0.6)	3.4 (0.8)
Neutral	Self	647 (94)	644 (131)	3.7 (0.7)	3.7 (0.6)
	Mother	739 (60)	699 (66)	3.6 (0.7)	3.5 (0.7)
	Acquaintance	789 (72)	759 (77)	3.2 (0.7)	3.4 (0.7)

#### Response Times

Given that hypotheses were formulated for the matching condition while nonmatching trials served as filler trials to make the task a useful task, a 2 (atimulus valence: *negative* vs. *neutral*) × 3 (association: *self* vs. *mother* vs. *acquaintance*) mixed-measures MANOVA with mean RTs as the dependent variable was conducted for the matching condition (for the use of MANOVA for analyzing repeated-measures designs, see O’Brien & Kaiser, 1985). This analysis revealed a significant main effect of the association, *F*(2, 36) = 49.20, *p* < .001, η_*p*_^2^ = .73, indicating faster responses in self-associated trials than in other-associated trials (Table 2). There was no main effect of stimulus valence, *F*(1, 37) = 1.80, *p* = .188, η_*p*_^2^ = .05, suggesting that mean RTs did not differ for the two samples. Importantly, there was no interaction of association and stimulus valence, *F*(2, 36) = 1.16, *p* = .325, η_*p*_^2^ = .06, suggesting that the effect of the association did not differ for the two stimulus-valence conditions. For a corresponding 2 (stimulus valence: *negative* vs. *neutral*) × 3 (association: *self* vs. *mother* vs. *acquaintance*) MANOVA in the hypothesis-irrelevant nonmatching condition, see Appendix B.

**Table 2:** Mean RTs (in ms) in matching trials as well as sensitivity in *d*′ in the main experiment as a function of stimulus valence, association, and block (standard deviations in parentheses)

Stimulus valence	Stimulus association	RTs		*d*′	
		Block		Block	
		First	Second	First	Second
Negative	Self	659 (87)	654 (89)	3.1 (0.8)	2.9 (0.9)
	Mother	681 (93)	679 (89)	2.9 (0.9)	2.7 (0.7)
	Acquaintance	727 (99)	715 (98)	2.7 (0.7)	2.6 (0.8)
Neutral	Self	609 (115)	626 (117)	3.1 (0.6)	3.1 (0.7)
	Mother	656 (106)	646 (94)	3.0 (0.6)	2.9 (0.7)
	Acquaintance	604 (123)	679 (113)	2.9 (0.6)	2.9 (0.8)

As specific hypotheses were formulated concerning the SPE, this effect was calculated. Based on previous results in the paradigm (see, e.g., Schäfer & Frings, 2019a; Sui et al., 2012; Woźniak & Knoblich, 2019), the SPE was calculated by subtracting mean RTs in the self-associated matching condition from the averaged RTs in the two non-self-associated matching conditions (i.e., mother- and acquaintance-associated matching condition).^2^ The overall SPE (*M* = 88 ms, *SD* = 81 ms) was significant, *t*(38) = 6.76, *p* < .001 (one-tailed), *d*_*Z*_ = 1.08. In the neutral condition, the SPE was *M* = 101 ms (*SD* = 76 ms), *t*(19) = 5.94, *p* < .001 (one-tailed), *d*_*Z*_ = 1.33; in the negative condition, the SPE was *M* = 74 ms (*SD* = 86 ms), *t*(18) = 3.74, *p* < .001 (one-tailed); *d*_*Z*_ = 0.86. As already indicated by the nonsignificant interaction of association and valence in the overall MANOVA, this SPE did not vary significantly in the two valence conditions, *t*(37) = 1.04, *p* = .153 (one-tailed), *d* = 0.33.

#### Accuracy

Accuracy rates were analyzed computing signal detection-sensitivity indices (*d*′) for each association condition. Correct responses in matching trials were considered hits, whereas erroneous responses in nonmatching trials were considered false alarms. We followed the log-linear approach to account for cases with 100% hits or 0% false alarms (Hautus, 1995; Stanislaw & Todorov, 1999) when computing the *d*′ indices. A 2 (stimulus valence: *negative* vs. *neutral*) × 3 (association: *self* vs. *mother* vs. *acquaintance*) mixed-measures MANOVA with *d*′ as the dependent variable revealed a significant main effect of association, *F*(2, 36) = 7.67, *p* = .002, η_*p*_^2^ = .30, indicating more sensitive responses in self-associated trials than in other-associated trials (Table 1). No further effects were significant, all *F*s < 1.

Again, we conducted analyses with the SPE (now for *d*′) as dependent variable. The overall SPE (*M* = 0.21, *SD* = 0.65) was significant, *t*(38) = 2.05, *p* = .024 (one-tailed), *d*_*Z*_ = 0.33. In the neutral condition, the SPE was *M* = 0.29 (*SD* = 0.71; *t*(19) = 1.85, *p* = .040 (one-tailed), *d*_*Z*_ = 1.33); in the negative condition, the SPE was *M* = 0.13, (*SD* = 0.58; *t*(18) = 0.95, *p* = .178 (one-tailed); *d*_*Z*_ = 0.22). The SPE did not vary significantly in the two valence conditions, *t*(37) = 0.80, *p* = .213 (one-tailed), *d* = 0.26. Thus, as for RTs, the SPE was numerically smaller in the negative condition; however, the difference was too small for finding a significant between-group effect.

#### Post Hoc Analyses

**Figure 1:**
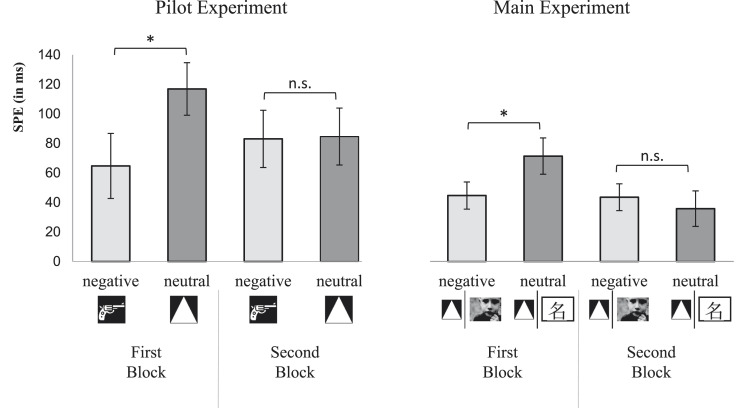
The SPE with neutral or negative stimuli in the first and second blocks in the pilot and main experiment [*p < .05 (one-tailed); error bars represent the standard error of the mean].

Based on the assumption that the effect of the negative connotation of the used stimuli might have decreased during the experiment simply because of the repeated exposure and, furthermore, because of the association with the self (constituting some kind of a self-protection mechanism), we added a post hoc analysis. Hence, regarding these assumptions, we set out to test whether the interaction of the SPE with stimulus valence might depend on the duration of the experiment. To test for the dependence of the SPE on stimulus valence separately in a first and second block of the experiment, we split the experiment in two blocks with 150 trials in each block. Next, we conducted a 2 (stimulus valence: *negative* vs. *neutral*) × 2 (block: *first* vs. *second*) mixed-measures ANOVA with the SPE_RT_ as the dependent variable. Importantly, the ANOVA revealed no significant main effect, both *F*s < 1.08, but a significant interaction of stimulus valence and block, *F*(1, 37) = 6.47, *p* = .015, η_*p*_^2^ = .15, indicating that the dependence of the SPE on stimulus valence differed between the two blocks. The difference between the SPE with negative stimuli and the SPE with neutral stimuli in the first block conformed to the hypothesis; it was associated with *t*(37) = 1.85, *p* = .036 (one-tailed), *d* = .59. A comparison of the SPEs in the second block showed no significant difference, |*t|* < 1 (Figure 1). However, the post hoc nature of the analysis makes adjustments necessary; thus, the difference in the first block missed the criterion of significance (i.e., *p* = .036 > .05/2).

With regard to accuracy, a 2 (stimulus valence: *negative* vs. *neutral*) × 2 (block: *first* vs. *second*) mixed-measures ANOVA with SPE_*d*′_ as the dependent variable revealed no significant effects, *F*(1, 37) = 3.53, *p* = .068, η_*p*_^2^ = .087 for block, F < 1 for valence, and *F*(1, 37) = 1.62, *p* = .211, η_*p*_^2^ = .042 for block × valence.

### Discussion

The pilot experiment revealed a significant SPE overall, suggesting that self-prioritization also worked with the weapon symbols and thereby indicating that the SPE can occur with various stimuli (which has been demonstrated by previous studies as well; e.g., Schäfer, Wesslein, et al., 2016; Stein et al., 2016; Woźniak, et al., 2018). Concerning the postulated moderation of the SPE by valence, the SPE with negatively connoted stimuli was numerically reduced (for RTs and accuracy) in comparison to the SPE with neutral stimuli (i.e., the typical SPE). Not unexpectedly (see the Participants section), however, the reduction was not large enough to be highly likely to produce a significant interaction effect given the size of our pilot sample.

Remarkably, the significant interaction with block suggested that a possible reduction of the SPE due to the valence of the to-be-integrated stimuli might be influenced by further processes. An exploratory post hoc analysis indicated that an overall reduction due to negative valence may have been hidden because the negativity of the stimuli vanished over the course of the experiment (as a difference was only indicated in the first half of the experiment).

Two assumptions gave reason to test for the effect of negative valence in a first and second block separately: first, a reduction of the negative valence of the stimuli due to repeated exposure and, second, a reduction due to the association of the stimulus with the self. According to the first assumption, the well-known mere-exposure effect (Zajonc, 1968) describes the phenomenon that mere repeated exposure of a person to a particular stimulus enhances the person’s attitude toward the stimulus. Meta-analyses leave little doubt that this effect is a robust, reliable phenomenon and highlight an even larger mere-exposure effect with brief exposure duration and after a fairly small number of exposures (about 10–20 stimulus presentations; Bornstein, 1989). Further evidence comes from a study in which the attention-grabbing effect of negative stimuli was found for the first presentation of the stimulus, but not for further presentations (Harris & Pashler, 2004), suggesting a diminishing of the effect of negative valence with repeated presentation.

According to the second assumption (of a possible reduction of negativity due to the association of a stimulus with the self), one can again refer to studies from the broader self-concept literature suggesting mechanisms that keep or reinstate a positive self-concept. In that regard, the well-established minimal-group phenomenon in social psychology (i.e., that the mere categorization of individuals into arbitrary social categories elicits in-group favoritism; Otten, 2016; Tajfel et al., 1971) can be reinterpreted as a self-concept process: Assigning a new yet unknown trait to a participant’s self leads to an instantaneously positive evaluation of this trait (Otten & Wentura, 1999, 2001; see also Wentura & Greve, 2005). Additionally, even if a negative characteristic becomes part of the self-concept, self-protective processes might neutralize this feature (Greve, 1990; Greve & Wentura, 2003, 2010; Wentura & Greve, 2005). Applying these considerations to the pilot experiment, the symbol of a bomb, for example, initially is a reference to a clearly negatively connoted weapon. After a while, due to the assignment of this symbol to the self, it might become less negative.

Regarding the methodological aspects of the pilot experiment, it is plausible that the influence of the negativity of the stimuli decreased over the course of the experiment so that an attenuation of the SPE was only found in the first block of trials. Yet, the assumption of an influence of block on the interplay of the SPE with valence was just formulated post hoc. Consequently, the power to detect the postulated effects (i.e., a difference due to stimulus valence in the first block and no such difference in the second block) in the pilot experiment was rather small. The main experiment reinvestigates exactly this dynamic of an attenuation of the SPE only in a first, but not in a second block with more power.

## Main Experiment

First of all, the main study aims at putting the finding of a block-modulation of the SPE reduction to another test – importantly with more power to detect the interaction of stimulus valence and block. Furthermore, while all previous studies concerning the integration of negative stimuli varied valence quasi-experimentally (i.e., by using materials with fixed, that is not experimentally manipulated valence) and thus by default opened the door to alternative explanations, a replication using an experimental modulation was considered to be informative. Hence, the same a priori neutral stimuli were used in both stimulus-valence conditions. Evaluative conditioning was used to either induce a negative connotation to these stimuli (in the negative condition) or not (in the neutral condition). The evaluative-conditioning procedure is used to change the liking for a stimulus simply by pairing it with a positive or negative stimulus (for a review, see De Houwer et al., 2001). Thus, aiming at an experimentally induced negative connotation of a priori neutral stimuli, we postulate the same data pattern such as in the pilot experiment: a reduction of the SPE with negative stimuli in the first half of the experiment, but not (or at least less) in the second.

### Method

#### Participants

Eighty-nine students from the University of Trier took part in the experiment receiving course credit. Data of five participants were discarded right after data collection, three participants because of a color blindness (note that the evaluative-conditioning method included a color-naming task, see below) and two participants because of technical errors. Furthermore, a distribution analysis for outlier performance scores (according to the parameters of Tukey, 1977) indicated that three participants committed far too many errors (i.e., extreme outlier values according to Tukey, 1977). The data of these three participants were discarded before the main analysis resulting in a final sample size of *N* = 81 (63 female; 42 participants in the negative-valence condition). The median age was 21 years (ranging from 18 to 31), and all had normal or corrected-to-normal vision.

Power analysis was oriented on two criteria. On the one hand, on the interaction effect of stimulus valence and block and, on the other hand, on the difference between the SPEs in the negative-valence and neutral conditions in the first block. In the pilot experiment, the interaction of stimulus valence and block was associated with η_*p*_^2^ = .15. To replicate this effect with power 1 − β = .80 (α = .05), a total sample size of *N* = 52 is needed (*n* = 26 participants in each group). Furthermore, the *t*-test comparing the SPEs in the first block was associated with *d* = .59. To replicate this effect with power 1 − β = .80 (α = .05, one-tailed), a total sample size of *N* = 74 is needed (*n* = 38 participants per group). Given the actual sample size of *N* = 81, the power to detect the relevant interaction was 1 – β = .95 and the power to detect the relevant difference between the two SPEs in the first block was 1 – β = .83 (all power calculation were run with G*Power; Faul et al., 2007).

#### Design

Inspired by the pilot experiment, the full experimental design comprised a 2 (stimulus valence: *negative* vs. *neutral*) × 2 (matching condition: *matching* vs. *nonmatching*) × 3 (association: *self* vs. *mother* vs. *acquaintance*) × 2 (block: *first* vs. *second*) mixed-measures design.

#### Material and Apparatus

Everything was the same as in the pilot experiment except that a triangle, a circle, and a square were used as to-be-associated stimuli in both stimulus-valence conditions and that four negative or four neutral pictures were used as stimuli for the evaluative-conditioning procedure. In more detail, pictures of Chinese signs were used as neutral pictures. For the negative stimuli, pictures were chosen based on valence and arousal ratings in a large sample study (Postzich et al., 2016); four pictures with moderate arousal and clearly negative valence rating were chosen (see Appendix C). These pictures were presented subtending a visual angle of 6.7° horizontally and 2.9° vertically.

#### Procedure

**Figure 2:**
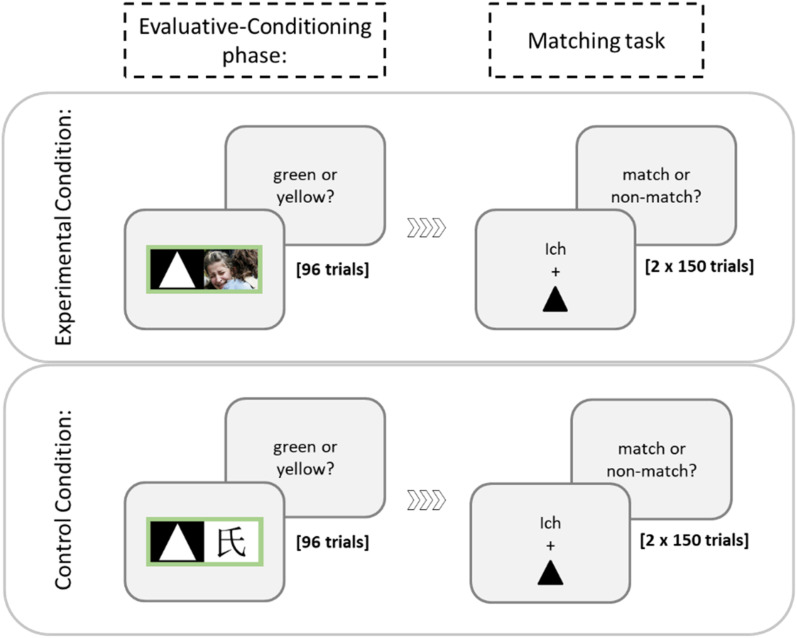
Schematic presentation of the procedure in the two stimulus-valence conditions in the main experiment: The only difference was the valence (either negative or neutral) of the pictures in the evaluative-conditioning phase.

The procedure was the same as in the pilot experiment except that an evaluative-conditioning procedure (see, e.g., Blask et al., 2016) preceded the matching paradigm. In a nutshell, participants worked through a simple color-naming task in which the three geometric shapes were presented together with either negative or neutral pictures. By the use of this procedure, the three geometric shapes (triangle, circle, and square) were predicted to acquire a negative connotation or remain neutral – depending on whether they were combined with negative or neutral pictures (which was varied between participants). Specifically, each trial started with the presentation of a blank screen for 500 ms, followed by a centered fixation cross for 500 ms. Then, an image was presented at the center of the screen for 1,000 ms in which one of the geometric shapes was presented with a negative (or neutral) picture within a colored frame (Figure 2). Participants were instructed to focus the center of the screen, and their task was simply to identify the color of the frame (i.e., N-key for a yellow and C-key for a green frame). A blank screen followed until response was given or until 500 ms elapsed, and then, the next trial started. Each participant worked through 10 practice trials, in which feedback was provided, and 96 test trials of this task. Half of the test trials included a yellow frame and half of them a green frame; each geometric shape was presented equally often with each frame (resulting in 16 trials for each shape–color combination). Trials were presented in a random order.

### Results

Only correct responses with RTs above 200 ms and below 1.5 interquartile ranges above the third quartile of the overall RT distribution (Tukey, 1977) were used for the RT analysis. Averaged across participants, 79% of the trials were selected for RT analysis; 17% of the trials were excluded because of erroneous responses and 4% due to the RT-outlier criteria. Mean RTs and sensitivity measures are shown in Table 2.

#### Response Times

Due to the fact that the nonmatching condition is only a filler condition to make the task a useful task, we conducted a 2 (stimulus valence: *negative* vs. *neutral*) × 3 (association: *self* vs. *mother* vs. *acquaintance*) × 2 (block: *first* vs. *second*) mixed-measures MANOVA in the matching condition with mean RTs as the dependent variable. This analysis revealed a significant main effect of the association, *F*(2, 78) = 43.41, *p* < .001, η_*p*_^2^ = .53, indicating an influence of the previously learned associations (Table 2). There was no other significant main effect, both *F*s < 2.64, both *p*s > .110. There was a significant interaction of association and block, *F*(2, 78) = 4.66, *p* = .012, η_*p*_^2^ = .11, suggesting that the effect of the association was different in the first and second halves of the experiment. Most important, the relevant three-way interaction of association, stimulus valence, and block was also significant, *F*(2, 78) = 3.43, *p* = .037, η_*p*_^2^ = .08. No other interactions were significant, all *F*s < 1, all *p*s > .765. For a corresponding 2 (stimulus valence: *negative* vs. *neutral*) × 3 (association: *self* vs. *mother* vs. *acquaintance*) × 2 (block: *first* vs. *second*) MANOVA in the hypotheses-irrelevant nonmatching condition, see Appendix B.

Again, to test for the hypothesis, the SPE was compared in the two valence conditions separately for the two blocks. The mean values of the SPE in RTs already confirmed the postulated data pattern from the pilot study (see Figure 1). A resulting 2 (stimulus valence: *negative* vs. *neutral*) × 2 (block: *first* vs. *second*) mixed-measures ANOVA emphasized this with a significant interaction of stimulus valence × block, *F*(1, 79) = 6.94, *p* = .010, η_*p*_^2^ = .08 (while valence did not cause an overall main effect, *F* < 1; the main effect of block was significant, *F*(1, 79) = 7.87, *p* = .006, η_*p*_^2^ = .09). The a priori planned comparison of the SPE with neutral and negative stimuli in the first block was significant, *t*(79) = −1.76, *p* = .042 (one-tailed), *d* = .41 (for the sake of completeness, note that this difference was – as hypothesized – not significant in the second block, *t* < 1; see Figure 1).

#### Accuracy

Accuracy rates were analyzed computing signal detection-sensitivity indices (*d*′). A 2 (stimulus valence: *negative* vs. *neutral*) × 3 (association: *self* vs. *mother* vs. *acquaintance*) × 2 (block: *first* vs. *second*) mixed-measures MANOVA with *d*′ as the dependent variable revealed a significant main effect of association, *F*(2, 78) = 11.82 *p* < .001, η_*p*_^2^ = .23, indicating more sensitive responses in self-associated trials than in other-associated trials. No further main effect or interaction was significant, *F*(1, 79) = 2.88, *p* = .094, η_*p*_^2^ = .04, for the main effect of block, all other *F*s < 1.48, all other *p*s > .228. For the sake of completeness, a 2 (stimulus valence: *negative* vs. *neutral*) × 2 (block: *first* vs. *second*) mixed-measures ANOVA with SPE_*d*′_ as the dependent variable revealed no significant effects, all *Fs* < 1). Thus, the RT result was not due to a speed accuracy trade-off.

### Discussion

The results of the main experiment replicated the data pattern, which was found in an exploratory post hoc analysis in the pilot experiment: an interaction of the effect of stimulus valence with experimental block. Again, a reduction of the SPE with negatively connoted stimuli was only indicated in the first half of the experiment, but not in the second half. Before further elaborating on these results, we present data collapsed for the two experiments.

## Comparison of the Experiments

To test for the homogeneity and robustness of the data pattern in the two experiments, we collapsed the data of both experiments. We first ran a mixed-measures ANOVA with the hypothesis-relevant factors stimulus valence (*negative* vs. *neutral*) and block (*first* vs. *second*) as well as the additional between-subject factor experiment (*pilot exp.* vs*. main exp.*) and with the SPE in RTs as the dependent variable. The factor experiment does not moderate any effect, all *F*s < 1. Therefore, we removed the factor experiment to get an overall result that does not give the pilot study (with its small sample size) the same weight as the main study. The overall interaction of stimulus valence and block as indicated by the two-way interaction was significant, *F*(1, 118) = 13.04, *p* < .001, η_*p*_^2^ = .099, emphasizing the postulated interplay of stimulus valence and block. Follow-up tests indicated a significantly smaller SPE with negative symbols compared to neutral ones in the first block, *t*(118) = −2.57, *p* = .006 (one-tailed), *d* = .47. No such difference was found in the second half, |*t|* < 1.

Additionally, we calculated Bayes factors (JZS default Bayes factor for *t*-tests; see Rouder et al., 2009, with a scale parameter of *r* = 1/√2) for the two essential results. First, the difference in SPE change from Block 1 to Block 2 for the two valence groups (i.e., the interaction effect reported above) was associated with a Bayes factor of BF_10_ = 57.7 (i.e., “very strong evidence” according to Jeffreys, 1961, Wagenmakers et al., 2011). Second, the test of the first block SPE being smaller in the negative condition compared to the neutral one for the two valence groups was associated with a Bayes factor of BF_+0_ = 7.3 (i.e., “substantial evidence” according to Jeffreys, 1961, Wagenmakers et al., 2011).

For accuracy, a mixed-measures ANOVA with the hypothesis-relevant factors stimulus valence (*negative* vs. *neutral*) and block (*first* vs. *second*) as well as the additional between-subject factor experiment (*pilot exp.* vs*. main exp.*) and with the SPE in *d*′ as the dependent variable yielded no moderations by the factor experiment, all *F*s < 1 except block × experiment, *F*(1, 116) = 2.18, *p* = .143, η_*p*_^2^ = .018. Again, we removed the factor experiment (see above). The final analysis yielded no significant effects, *F*(1, 118) = 1.46, *p* = .230, η_*p*_^2^ = .012 for block, *F* < 1 for valence, and *F*(1, 118) = 1.65, *p* = .201, η_*p*_^2^ = .014 for block × valence. Thus, although we cannot confirm the results for RTs, there is no indication of a speed accuracy trade-off.

## General Discussion

We set out to put the assumption of a *functional self-prioritization* to another test and to further specify it. In two experiments, we compared the integration of negatively connoted stimuli with the integration of neutral stimuli, using different variants of potentially negative stimuli (including a full experimental manipulation of this factor in the main experiment). The results did not indicate an overall reduction of the SPE due to negative stimulus valence. However, in both experiments, we found a reduced SPE for RTs in the first block of trials; this difference vanished in the second block.

The results support important assumptions about the minimal self. First, the emergence of the SPE even with negative stimuli (note that it was reduced, but not eliminated) suggests that the integration of stimuli into the self-network – once they are perceived as being self-relevant (in this case, due to mere instruction) – is unavoidable. This integration of stimuli although negatively connoted could be seen as a counter argument against the functionality of the minimal self. On the other hand, this indication of an unavoidable formation of self-associations suggests an inevitable component of such association formations: The pure co-occurrence of a neutral stimulus with a self-relevant label results in an integration of this stimulus. Might the functionality of self-associations be the way that the formation of self-associations is a very fundamental process, some kind of an essential preset, but it nevertheless adapts partly to contextual aspects (like the valence of the to-be-associated content)? However, the partly unavoidable integration of stimuli indicates a commonality with processes of simple feature bindings as with the integration of stimuli (and also responses) with each other, and theories explicitly postulate that the formation of these bindings is automatic (Henson et al., 2014; Hommel, 1998; for a recent framework integrating theories about feature binding and retrieval, see Frings et al., 2020). Hence, the partly unavoidable self-integration contributes to the ongoing debate on whether concepts of feature bindings can be transferred to the representation of people (oneself and others; for a current theoretical review, see Hommel, 2019). At the very least, the finding that self-integration is partly unavoidable constitutes a commonality of self-perception and object perception (Hommel, 2019).

Second, the results indicate an undeniable effect of negative valence on self-associations which, however, is modulated by further mechanisms. One might consider that the standard SPE – meaning the SPE with neutral stimuli – reduces somehow over the course of the experiments (Figure 1). It remains a question for further research to investigate the development of the SPE over time, but at this point, it also indicates an effect of stimulus valence as the SPE with negative stimuli develops differently. While the SPE with neutral stimuli decreases over the course of the experiment, the SPE with negative stimuli increases – it perhaps just comes to a standard level. Hence, the data do not support an overall reduction of self-associations with stimuli, which are supposed to be negative. Rather, a functional reduction of negative self-associations seems to occur under specific circumstances. The different effect of valence on the SPE throughout the experiment (or the different development of the SPE over the course of the experiment depending on the valence of the to-be-integrated stimuli) suggests that the effect of valence on the SPE is sensible to dynamics over the time.

The depending reduction of the SPE due to negative valence of the stimuli emphasizes one thing rather clearly: the *protection mechanism* of self-association. Considering the previously mentioned, wide-ranging effects of self-relevance on everyday perception and processing, this is an important conclusion regarding the further understanding of the minimal self. This assumption of a functionality and, more specifically, a self-serving bias of self-associations, is in line with previous research about a positivity bias for self-related content (e.g., Greenwald, 1980; Kuiper & Derry, 1982; Taylor & Brown, 1988).

This reminiscence of the classic self-serving literature is rather unspecific with regard to potential base mechanisms that finally *serve the self* in our experimental setting. A candidate for such a base mechanism is the affective matching process (Klauer & Stern, 1992; see also Klauer & Musch, 2002; Wentura, 2000). If two valent stimuli (e.g., *Einstein* and *fond of animals*) are presented together in the context of a binary decision task with responses having the character of affirmation versus negation (e.g., “Does the stimulus pair consist of a name and an adjective?”), the evaluations of the two stimuli are automatically processed *and* automatically compared in a spontaneous plausibility check, resulting in a tendency to affirm or negate. Thus, if they match in valence (as is the case for *Einstein* and *fond of animals*) an affirmative answer is facilitated; if they do not match, however (e.g., *Hitler* and *fond of animals*), the conflict slows down an affirmative answer. This resembles the situation in our experiments: Participants should affirm the arbitrary assignment of shape and label. If the shape is negative and the label is positive (as can be assumed for the self-label of the average participant), the affirmation is slowed down compared to the control condition with neutral labels.^3^ Thus, this process can explain the empirical result of a reduced SPE (in the first block) of the matching task. However, at a more abstract level, the process provides a kind of resilience to bind a negative attribute to the (positively valenced) self if the parallel occurrence of attribute and a self-reference stimulus results in a spontaneous “nope [they do not go together].”

Whether it is the vanishing of the negativity of simple weapon symbols over the time (e.g., due to mere exposure, Zajonc, 1968) or whether it is the increasingly strong assignment of the stimulus to the self, which then prevents a reduction in the second block needs to be content of further research. The latter would additionally emphasize the postulated self-protection mechanism.

Taken together, the current findings strengthen previous results about a reduced integration of negative stimuli with the self and thereby further support the assumption of a highly functional minimal self. This minimal self can be seen as a network of associations, which are not only functional because they are specific (thereby guiding cognitive resources in a target-orientated fashion) and stable (thereby avoiding costly constant regenerations of associations) but also for two further reasons: On the one hand, the association of a negative stimulus with the self is smaller than the association of a neutral stimuli with the self, thereby indicating a prevention mechanism; on the other hand, associations with a negative stimulus are not completely prevented, contradicting a simple blind spot for negative (but potentially realistic) things.

## Figures and Tables

**Figure A1:**
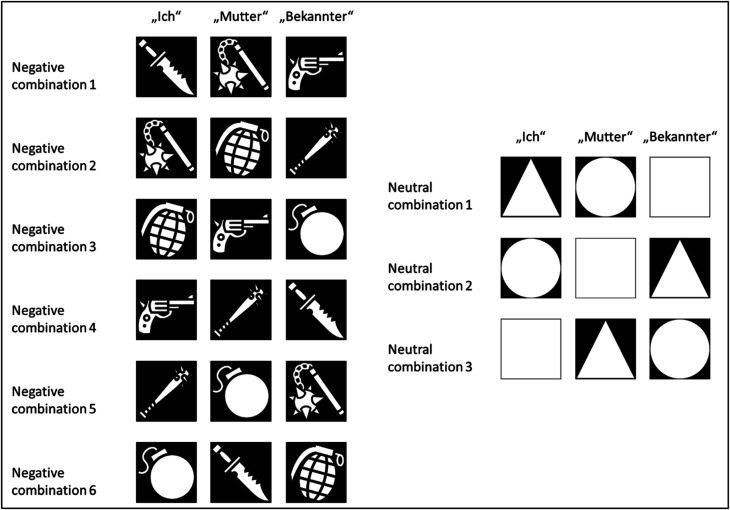
The negative and neutral stimulus combinations in the pilot experiment. Note that, in the negative-valence condition, one of these possible negative combinations was randomly chosen for each participant while in the neutral stimulus-valence condition, the three possible combinations were varied in a Latin-square design.

## Footnotes

^1^
Note that the study by Constable et al. (2020) and ours were conducted in parallel. However, we can identify differences in the studies that make our studies a valuable follow-up to the results from Constable and colleagues without criticizing them – besides the value of two completely independent realizations of a basic hypothesis resulting in comparable outcomes.

^2^
Note that it is inherent to the MANOVA approach for repeated measures to transform three within-participants conditions into two orthogonal contrasts, for example, a Helmert contrast: (a) first versus average of the second and third conditions and (b) second versus third condition. In this regard, the analysis of the SPE is equivalent to the a priori planned first Helmert contrast and therefore a follow-up to the overall MANOVA (Schäfer et al., 2017).

^3^
In addition, Klauer and Stern (1992) predicted and found that if the same stimulus pairs have to be negated (for the example: “Is it a name/name or adjective/adjective pair?”), the pattern of RTs reverses (i.e., *Einstein* and *fond of animals* is now slower than *Hitler* and *fond of animals*). There is no direct equivalent to this in our paradigm. However, in our setting one might expect that nonmatching (i.e., negation) responses to self-label trials are *faster* in the negative condition compared to the neutral condition. There is indeed a hint to such an effect in the main experiment (see Appendix B).

## Notes

**Open Data:** The following DOI generated by a public data repository pointing to the raw data underlying the findings reported in the article. Data are available at https://osf.io/mhx9u (Wentura et al., 2023)

## Appendix B

### Results for Non-Matching Trials

#### The Pilot Experiment

A 2 (stimulus valence: *negative* vs. *neutral*) × 3 (association: *self* vs. *mother* vs. *acquaintance*) mixed-measures MANOVA with mean RTs as the dependent variable in the hypothesis-irrelevant nonmatching condition (Table B1) revealed a significant main effect of the association, *F*(2, 36) = 49.20, *p* < .001, η_*p*_^2^ = .48, indicating fastest responses in trials with the self-relevant label (and one of the two non–self-relevant symbols). Furthermore, there was a significant interaction of this effect with stimulus valence, *F*(2, 36) = 4.04, *p* = .026, η_*p*_^2^ = .18, suggesting that the effect of the association was different in the two stimulus-valence conditions. There was no main effect of stimulus valence, *F*(1, 37) = 2.11, *p* = .155, η_*p*_^2^ = .05, suggesting that mean RTs did not differ for the two samples.

**Table B1:** Mean RTs in nonmatching trials in the pilot experiment and the main experiment as a function of stimulus valence and association (standard deviations in parentheses)

Stimulus valence	Stimulus association (label)	Exp. 1	Exp. 2	
			Block 1	Block 2
Negative	Self	800 (75)	762 (93)	748 (98)
	Mother	797 (78)	765 (103)	743 (96)
	Acquaintance	846 (103)	756 (91)	752 (99)
Neutral	Self	752 (75)	743 (131)	715 (122)
	Mother	785 (63)	732 (121)	727 (114)
	Acquaintance	801 (79)	719 (125)	711 (123)

#### The Main Experiment

A 2 (stimulus valence: *negative* vs. *neutral*) × 3 (association: *self* vs. *mother* vs. *acquaintance*) × 2 (block: *first* vs. *second*) mixed-measures MANOVA in the hypotheses-irrelevant nonmatching condition revealed a significant main effect of block, *F*(1, 79) = 5.43, *p* = .022, η_*p*_^2^ = .06, but no other significant effects (neither one of the main effects nor the interaction), all *F*s < 1.75, all *p*s > .180 (Table B1).

We add here an exploratory analysis that deserves attention with regard to a suggestion made in the *General Discussion* section. We analyzed the SPEs in a 2 (stimulus valence: *negative* vs. *neutral*) × 2 (type of trial: *matching* vs. *nonmatching* ) × 2 (block: *first* vs. *second*) ANOVA. We found a significant three-way interaction, *F*(1, 79) = 7.86, *p* = .006, η_*p*_^2^ = .090. Decomposing this interaction showed that Block 1 yielded a two-way interaction, *F*(1, 79) = 5.96, *p* = .017, η_*p*_^2^ = .070, which is completely missing in Block 2, *F* < 1. The significant two-way interaction for *matching* trials in Block 1 – that is, the reduced SPE in the negative condition compared to the neutral condition – was already reported in the main text. For *nonmatching* trials, numerically the pattern reversed, *F*(1, 79) = 3.67, *p* = .059, η_*p*_^2^ = .044, that is, the SPE is *larger* for the negative condition. This overall pattern fits to the assumption of the affective matching theory (Klauer & Stern, 1992; see the main text for elaboration) that to *affirm* a negative shape/self-label pair is slowed down compared to the control condition, but that to *negate* such a stimulus pair is facilitated. We emphasize the post hoc character of this analysis; the experiment was not planned to test this affective matching hypothesis. Moreover, the pattern was not found in the pilot experiment.

## Appendix C

### Materials of the Main Experiment

**Table C1:** Information about the four negative pictures which were selected as stimuli for the negative condition in the evaluative-conditioning procedure: valence and arousal ratings according to Postzich et al. (2016) with negative values on valence rating indicating negative valence in comparison to positive valence and negative ratings on arousal rating indicating calmness in comparison to activation, ranging from −100 to +100

Description in Postzich et al. (2016)	Valence rating	Arousal rating
“Females cry”	−49.43	11.94
“Person alone”	−64.94	12.40
“Family homeless“	−68.82	26.57
“Child sad”	−53.77	8.66
